# Ancistrocladinium A Induces Apoptosis in Proteasome Inhibitor-Resistant Multiple Myeloma Cells: A Promising Therapeutic Agent Candidate

**DOI:** 10.3390/ph16081181

**Published:** 2023-08-18

**Authors:** Daniela Brünnert, Raina Seupel, Pankaj Goyal, Matthias Bach, Heike Schraud, Stefanie Kirner, Eva Köster, Doris Feineis, Ralf C. Bargou, Andreas Schlosser, Gerhard Bringmann, Manik Chatterjee

**Affiliations:** 1 University Hospital of Würzburg, Comprehensive Cancer Center Mainfranken, Translational Oncology, 97080 Würzburg, Germanychatterjee_m@ukw.de (M.C.); 2 Institute of Organic Chemistry, University of Würzburg, 97074 Würzburg, Germany; 3 Department of Biotechnology, School of Life Sciences, Central University of Rajasthan, Bandar Sindri, Kishangarh 305817, India; pankaj_bio@curaj.ac.in; 4 Rudolf Virchow Center for Experimental Biomedicine, University of Würzburg, 97080 Würzburg, Germany

**Keywords:** multiple myeloma, ancistrocladinium A, naphthylisoquinoline alkaloids, proteasome inhibitor resistance, RNA splicing, cellular stress response, proteasome subunit beta type-5 (PSMB5), activating transcription factor 4 (ATF4), ataxia teleagiectasia mutated (ATM), H2A histone family member X (H2AX)

## Abstract

The *N*,*C*-coupled naphthylisoquinoline alkaloid ancistrocladinium A belongs to a novel class of natural products with potent antiprotozoal activity. Its effects on tumor cells, however, have not yet been explored. We demonstrate the antitumor activity of ancistrocladinium A in multiple myeloma (MM), a yet incurable blood cancer that represents a model disease for adaptation to proteotoxic stress. Viability assays showed a potent apoptosis-inducing effect of ancistrocladinium A in MM cell lines, including those with proteasome inhibitor (PI) resistance, and in primary MM cells, but not in non-malignant blood cells. Concomitant treatment with the PI carfilzomib or the histone deacetylase inhibitor panobinostat strongly enhanced the ancistrocladinium A-induced apoptosis. Mass spectrometry with biotinylated ancistrocladinium A revealed significant enrichment of RNA-splicing-associated proteins. Affected RNA-splicing-associated pathways included genes involved in proteotoxic stress response, such as PSMB5-associated genes and the heat shock proteins HSP90 and HSP70. Furthermore, we found strong induction of ATF4 and the ATM/H2AX pathway, both of which are critically involved in the integrated cellular response following proteotoxic and oxidative stress. Taken together, our data indicate that ancistrocladinium A targets cellular stress regulation in MM and improves the therapeutic response to PIs or overcomes PI resistance, and thus may represent a promising potential therapeutic agent.

## 1. Introduction

Multiple myeloma (MM), the second most common hematological cancer [1], is characterized by the expansion of malignant plasma cells producing non-functional immunoglobulins and causing hematopoietic impairment, osteolytic bone destruction, and kidney injury [2]. Because of immunoglobulin synthesis and increased amounts of misfolded proteins, MM cells harbor high proteotoxic stress levels [3] requiring up-regulation of various coping mechanisms including proteasomal activity or the unfolded protein response (UPR) [4]. In this context, the clinical introduction of proteotoxic stress-targeting agents such as the proteasome inhibitors bortezomib [5], ixazomib [6], or carfilzomib [7] and the histone deacetylase inhibitor (HDAC) panobinostat [8] marked a new era of MM therapy. MM has, therefore, become a model disease for the dependence of transformed cells on coping mechanisms to adapt to proteotoxic stress. However, MM still remains largely incurable, and patients inevitably suffer from relapses due to the development of drug resistance. Resistance mechanisms include ABCB1 (MDR1) overexpression upon carfilzomib treatment [9] or PSMB5 mutations resulting from bortezomib [10] or ixazomib therapy [11]. Therefore, further development of new drugs to improve PI treatment or overcome PI resistance remains a challenging task.

Precursor messenger RNA (pre-mRNA) alternative splicing has been reported to contribute to malignant transformation [12,13]. It can result in intron retention, exon skipping, alternative donor or acceptor site selection, and complex events with more than one splicing alteration [14]. Thus, targeting RNA splicing has been characterized as a promising potential anti-cancer treatment strategy [15,16]. In MM, a high number of novel splice loci was shown to be associated with poor survival, suggesting that alternative splicing might represent a potential driver mechanism [17]. In line with this assumption, the knockdown of the serine- and arginine-rich splicing factor 1 (SRSF1) was shown to inhibit cell survival and proliferation in MM cells [18]. In addition, the upregulation of SRSF1 has recently been found to be regulated by the transcription factor YY1, a pro-oncogenic driver in MM [19]. Furthermore, the spliceosome was identified as a therapeutic vulnerability under proteasome inhibition, supporting the idea of spliceosome targeting as a potential therapeutic strategy in MM [20].

**Figure 1:**
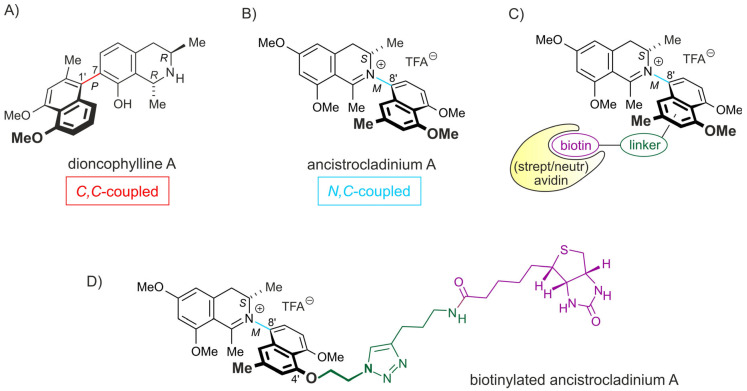
Chemical structures of (**A**) dioncophylline A, (**B**) ancistrocladinium A, (**C**,**D**) biotinylated ancistrocladinium A as (**C**) the general derivative and the schematic labeling with biotin and its interaction with streptavidin, and (**D**) the specific, here prepared and used biotin-labeled analog of ancistrocladinium A.

*C,C*-coupled naphthylisoquinoline (NIQ) alkaloids such as dioncophylline A (Figure 1A) are an emerging class of structurally, biosynthetically, and pharmacologically unique natural products. They have been isolated from tropical lianas like *Triphyophyllum peltatum* and from the Congolese liana *Ancistrocladus ileboensis* and show anti-parasitic and antitumor activities [21,22].

We previously demonstrated strong cytotoxic effects of *C,C*-coupled naphthylisoquinoline alkaloids on the MM cell line INA-6, the drug-sensitive acute lymphoblastic CCRF-CEM leukemia cell line, and its multidrug-resistant subline, CEM/ADR5000. Remarkably, the cytotoxicity of *C,C*-coupled naphthylisoquinoline alkaloids was even stronger than that of the standard anti-MM agent drug melphalan in INA-6 MM cells [21]. Because of these encouraging results, we analyzed the cytotoxic properties of additional representatives of this class of metabolites. Within this group of natural products, the *N,C*-coupled naphthylisoquinoline alkaloids, equipped with an unprecedented chiral *N*-iminium aryl axis and a permanent positive charge, became of great interest due to their thrilling stereochemical implications and their remarkable biological activities [23]. Especially, ancistrocladinium A (Figure 1B), isolated from a Congolese *Ancistrocladus* liana, has anti-parasitic activities against *Plasmodium falciparum*, *Trypanosoma brucei rhodesiense*, and *Leishmania donovani*, without affecting normal cells, such as macrophages and rat myeloblasts [24]. However, the possible effect of ancistrocladinium A on human cancer, including MM, is yet unknown.

Therefore, we here explored whether ancistrocladinium A might affect the survival and pathobiology of MM cells.

## 2. Results

### 2.1. Treatment with Ancistrocladinium A Shows Activity against MM Cells, including PI-Resistant MM Cells, but Not in Non-Malignant Peripheral Blood Mononuclear Cells (PBMCs)

**Figure 2:**
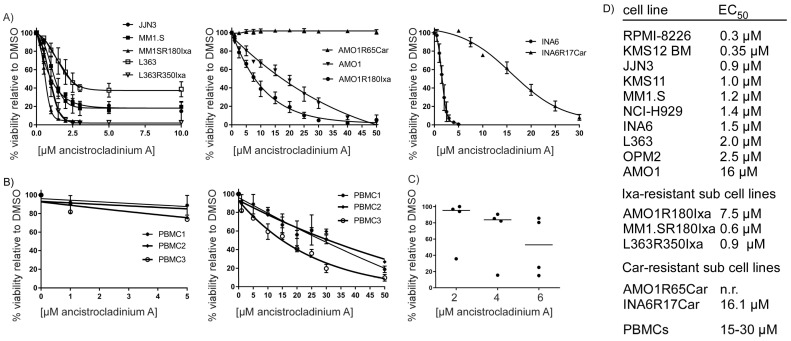
Ancistrocladinium A induces apoptosis in MM cells. Results of viability analyses upon treatment of MM cells and non-malignant PBMCs with ancistrocladinium A are shown either as dose-response curves (**A**,**B**) or as a dot blot (**C**). Human MM cell lines, including PI-resistant sub-cell lines (**A**), PBMCs of three healthy donors (**B**), or primary CD138^+^ MM cells from four patients (**C**), were treated for 3 d with different concentrations of ancistrocladinium A prior to viability analysis by flow cytometry-based annexin V/propidium iodide staining. The respective EC_50_ values of all analyzed cell lines are summarized in the table (**D**). Three to five independent experiments were performed for MM cell line experiments.

First, we asked whether concentration-dependent treatment with ancistrocladinium A might specifically affect the survival of MM cells (Figure 2). Therefore, we analyzed the cellular viability of various human MM cell lines, including well-established PI-resistant cell lines, primary MM cells freshly obtained from diagnostic specimens of patients, and peripheral blood mononuclear cells (PBMCs) of healthy donors as a control upon treatment with different ancistrocladinium A concentrations for 3 d. We observed a decrease in viability by induction of apoptosis in all the MM cell lines, although to a different degree. While the majority of the MM cell lines were highly sensitive towards ancistrocladinium A treatment, with EC_50_ values between 0.2 and 3 µM (Figure 2A,D), AMO1 was less sensitive as indicated by an EC_50_ value of 16 µM (Figure 2A,D).

Next, we analyzed whether ancistrocladinium A might also be effective against five MM sub-cell lines with acquired resistance to the proteasome inhibitors (PI) ixazomib (Ixa) and carfilzomib (Car) (Figure 2A). We found that out of three Ixa-resistant cell lines, two cell lines (MM1.SR180Ixa and L363R350Ixa) were sensitive with an EC_50_ value of approximately 0.6–0.9 µM, whereas the third cell line, AMO1R180Ixa, remained less sensitive with an EC_50_ value of 7.5 µM. Interestingly, all ixazomib-resistant cell lines were even more (approximately 2-fold) sensitive than their parental cell lines. In contrast, the cellular viability of INA6R17Car (EC_50_ 16.1 µM) and AMO1R65Car (not reached EC_50_) was not substantially affected by ancistrocladinium A, indicating that the carfilzomib-resistant cell lines are also resistant against ancistrocladinium A.

In addition to MM cell lines, we also explored the effect of ancistrocladinium A on the survival of primary MM cells. CD138^+^ cells were isolated from diagnostic bone marrow specimens of four MM patients prior to treatment with three different concentrations of ancistrocladinium A for 3 d (Figure 1C,D). We found that ancistrocladinium A treatment led to a viability decrease at concentration levels between 4 and 6 µM (Figure 2C). In contrast to malignant MM cells, the viability of non-malignant PBMCs derived from three healthy donors was unaffected by ancistrocladinium A up to a concentration of 5 µM, indicating an MM-specific cytotoxic effect (Figure 2B left panel). Moreover, we analyzed higher concentrations of ancistrocladinium A and found that it affects the viability of PBMCs only at very high concentration levels ranging between 15 and 30 µM as EC_50_ values (Figure 2B right panel). Taken together, our data suggest that ancistrocladinium A might be an MM-specific therapeutic drug overcoming some forms of PI resistance.

### 2.2. Ancistrocladinium A Targets the RNA Splicing Machinery

To explore its mode of action, we used biotin-labeled ancistrocladinium A (Figure 1D) to pull down ancistrocladinium A-interacting proteins with high-affinity chromatography followed by nanoLC-MS/MS analysis. For the biotinylation of ancistrocladinium A, the biotin entity was covalently linked via the oxygen function at C-4′ (Figure 1B, Supplementary Figure S1, and Section 2.1 and Section 2.2).

**Figure 3:**
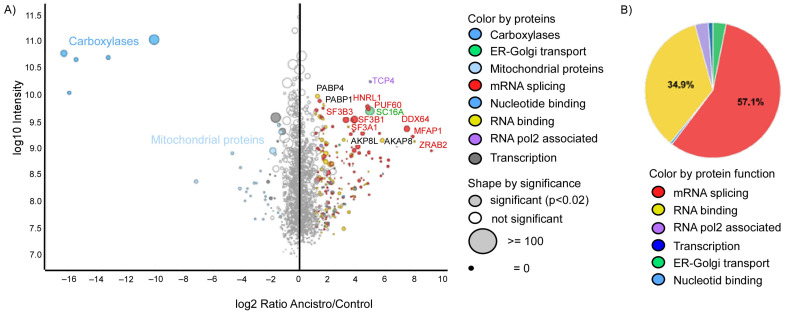
Identification of ancistrocladinium A-interacting proteins in INA-6 cell lysates. (**A**) Significantly enriched proteins are represented by gray-filled circles. Protein ratios and intensities are median values derived from three different replicates. The size of the circles correlates with the number of identified razors and unique peptides. Identified proteins are grouped according to their function, and each group is marked by a different color. Carboxylases have biotin as a prosthetic group and were, therefore, enriched specifically by the streptavidin beads, which were used as a control. Statistical significance was calculated using the Benjamini–Hochberg method (adjusted limma *p*-value < 0.02). (**B**) Pie chart showing the distribution of the different functional groups of significantly enriched proteins.

A total of 79 proteins were specifically enriched by the affinity pulldown approach with the biotinylated ancistrocladinium A as compared to streptavidin beads as a control (Figure 3A; Supplementary Table S1; Supplementary Figure S2). Among the enriched proteins, 57.1% belong to the mRNA splicing machinery, and 34.9% were identified as RNA-binding proteins (Figure 3B). RNA splicing-associated proteins include the splicing factor 3 (SF3) subunits (SF3A1, SF3A2, SF3A3, SF3B2, SF3B3, SF3B6) and the splicing factor 1 (SF1) (Supplementary Table S1). Other RNA splicing-associated proteins included the five serine- and arginine-rich splicing factors (SRSFs), namely SRSF2, SRSF3, SRSF7, SRSF9, and SRSF10, and various heterogeneous nuclear ribonucleoproteins (HNRNPs). In addition, factors involved in the regulation of transcription, endoplasmic reticulum/Golgi transport, nucleotide binding, and mitochondrial function have been identified. Of note, the carboxylases are only enriched in the streptavidin control beads and serve as a control for a successful assay.

### 2.3. Ancistrocladinium A Differentially Regulates the RNA Binding and RNA Splicing Associated Genes

Next, we asked whether the binding of ancistrocladinium A with the RNA splicing factor proteins might change the expression of alternative splicing gene profiles. We treated INA-6 cells for 4 h with ancistrocladinium A and subsequently performed RNA expression profiling using the Clariom D chip. We observed that 1103 genes, including pseudogenes, were upregulated, and 184 genes (and pseudogenes) were downregulated in the ancistrocladinium A-treated cells (Supplementary Figure S3A, Supplementary Table S2). Data interpretation by GSEA revealed that RNA processing was the most prominent biological theme. Furthermore, genes harboring the motif of the transcription-associated genes GTF2A2, SNAPC4, and GTF2E2 were also highly significantly enriched. Of note, splicing-associated pathways like the formation of quadruple SL U4, U5, U6 SNRNP, and spliceosomal tri-SNRNP complex AS assembly were also among the top 10 enriched pathways (Table 1). These data confirm the MS data that ancistrocladinium A targets RNA processes like RNA binding and splicing and transcription at the RNA level.

### 2.4. Ancistrocladinium A Induces RNA Splicing

Because RNA splicing-associated proteins were significantly enriched, we next sought to analyze splicing alterations in INA-6 cells upon ancistrocladinium A treatment using the human Clariom D chip. We observed a significant effect on the splicing of 747 genes, including 397 intron retentions, 137 alternative 5′ donor sites and 110 alternative 3′ acceptor sites, 80 cassette exons, 18 complex events comprising more than one splicing event in a gene, 3 alternative first exons, and 2 alternative last exons (Supplementary Figure S3B, Supplementary Table S3).

To gain more insight into potential RNA splicing-induced pathways, data were further interpreted by GSEA. We found that PSMB5 target genes and GTF2A2 target genes were among the top 10 enriched pathways. These genes contain at least one binding site for the target gene in the promotor region. Furthermore, cell cycle-associated genes were also found to be alternatively spliced, and in INA-6 cells, there was a trend towards a lower G0/G1 phase after 24 h treatment with ancistrocladinium A (Table 2; Supplementary Figure S4). These data indicate that ancistrocladinium A modulates the major RNA polymerase function as well as cell cycle genes.

### 2.5. Ancistrocladinium A Induces ATF4 Protein Expression

**Figure 4:**
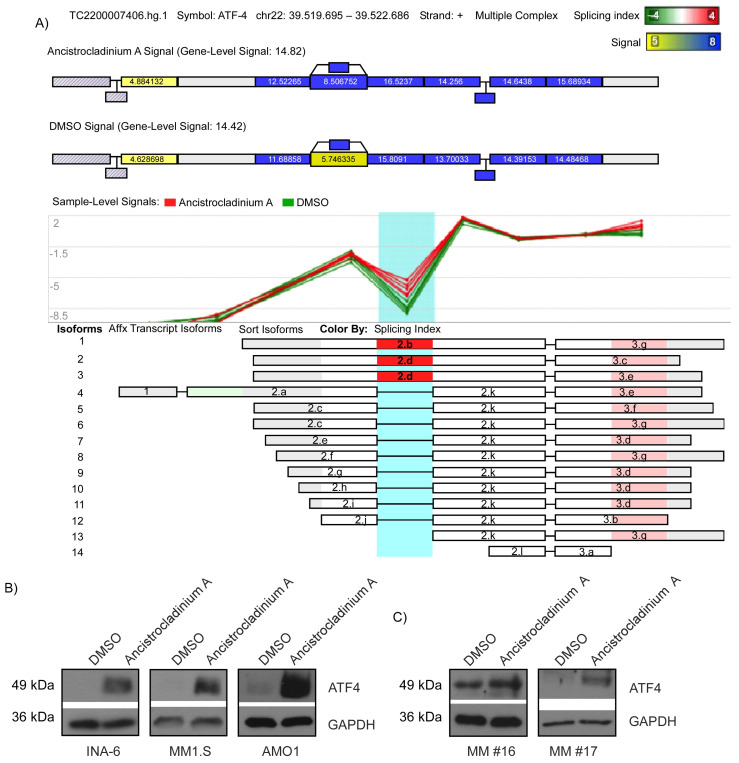
Ancistrocladinium A induces ATF4 protein expression in INA-6 cells. (**A**) Splicing effect on ATF4-RNA 4 h after treatment of INA-6 cells with ancistrocladinium A (**B**) INA-6, MM1.S, AMO1 cells and (**C**) two primary CD138^+^ MM cell samples were treated for 4 h with 3 µM ancistrocladinium A followed by protein lysis and Western blot analysis of ATF4 or GAPDH, which served as a loading control.

Because coping with proteotoxic stress is considered to represent a therapeutic Achilles’ heel for MM, we focused on PSMB5 as a critical part of the proteasome [25]. One key factor for this is the Activating Transcription Factor 4 (ATF4), which is induced during unfolded protein response (UPR) by an alternative splicing variant resulting in elevated protein expression [26]. We identified ATF4 amongst the genes with an alternative splicing (Figure 4A, Supplementary Figure S5). To check whether ancistrocladinium A can induce ATF4 protein expression, we treated INA-6, MM1.S, AMO1 (Figure 4B) and primary MM cells (Figure 4C) with ancistrocladinium A for 4 h prior to Western blot analysis and observed strongly induced expression of ATF4 protein. These data suggest that ancistrocladinium A might have a proteotoxic stress effect on MM cells.

### 2.6. Ancistrocladinum A Induces H2A.X Phosphorylation

**Figure 5:**
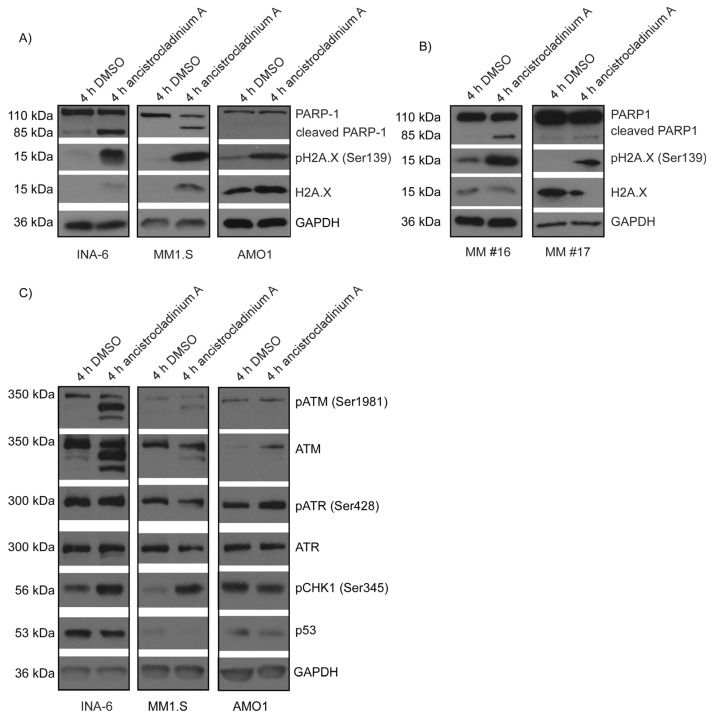
Ancistrocladinium A induces H2A.X phosphorylation. (**A**,**C**) INA-6, MM1.S, AMO1 cells, and (**B**) two primary MM cell samples were treated for 4 h with 3 µM ancistrocladinium A followed by protein lysis and Western blot analysis of H2X.A expression and phosphorylation, and of PARP cleavage. (**C**) INA-6, MM1.S, and AMO1 cells were analyzed for DNA damage response proteins ATM, ATR, and its phosphorylation sites, p53 and phosphorylation of CHK1. Expression analysis of GAPDH served as a loading control.

Because several histones and histone clusters were found in the ancistrocladinium A-induced gene expression profile suggesting DNA damage and subsequent oxidative stress induction, we next examined H2A.X phosphorylation on residue Ser139, which is a marker for DNA damage. We found a strong increase in H2A.X phosphorylation in INA-6 and MM1.S cells (Figure 5A) as well as in a primary MM cell sample (Figure 5B), along with PARP1 cleaving. In ancistrocladinium A insensitive AMO1 cells, on the contrary, induction of phosphorylation by ancistrocladinium A was only weak and did not result in cleavage of PARP1 (Figure 5A, Supplementary Figure S6).

### 2.7. Concomitant Treatment with Ancistrocladinium A Enhances the Apoptotic Effects of the HDAC Inhibitor Panobinostat

**Figure 6:**
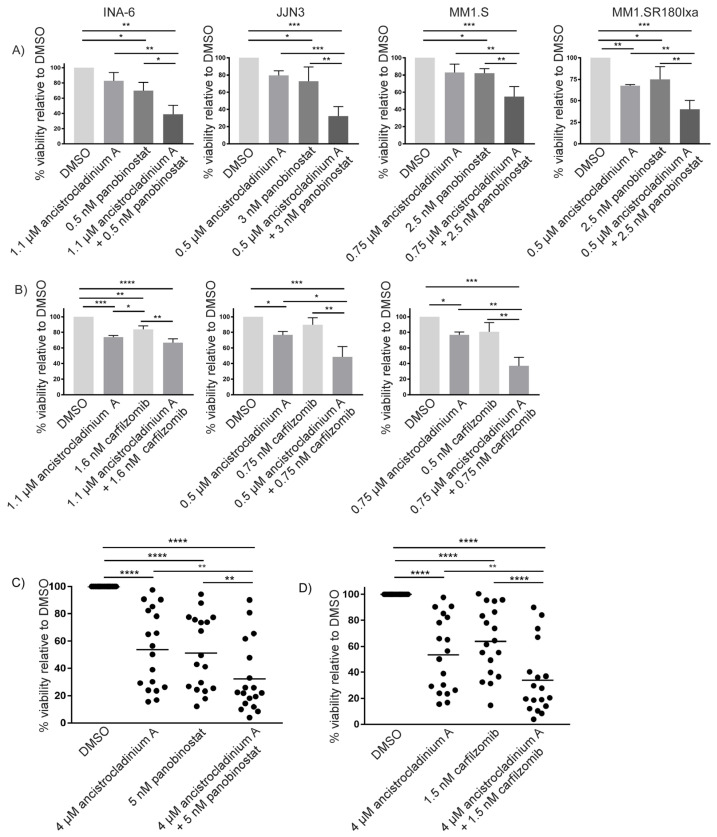
Combination with ancistrocladinium A enhances the apoptotic effects of panobinostat or carfilzomib on primary MM cells. The cell lines INA-6, JJN3, MM1.S, and MM1.SR180Ixa were treated for 3 d with EC_25_ concentrations of (**A**) ancistrocladinium A, panobinostat or a combination of both, (**B**) ancistrocladinium A, carfilzomib or a combination of both for 3 d followed by flow-cytometry-based viability analysis using an annexin V/propidium iodide staining assay. (**C**,**D**) Eighteen primary CD138+ MM cell samples, co-cultured on BM stromal cells, were treated with 4 µM ancistrocladinium A, 5 nM panobinostat, or 1.5 nM carfilzomib alone, or with a combination approach either with of ancistrocladinium A and panobinostat or ancistrocladinium A and carfilzomib for 3 d followed by viability analysis. DMSO treatment served as a solvent control, and results were normalized to the respective DMSO control. To statistically interpret the significance of the data, a 2-way ANOVA test followed by a Tukey’s multiple comparisons test was performed (* *p* < 0.05; ** *p* < 0.01; *** *p* < 0.001; **** *p* < 0.0001).

Another question was whether a combination approach of ancistrocladinium A with the clinically approved anti-MM agent panobinostat, an HDAC inhibitor [8], would result in enhanced cell death in MM cell lines when using EC_25_ doses of both compounds for 3 d. By conducting viability analyses, we found that this combination approach was effective in all four cell lines tested. Interestingly, the strongest combinatory anti-MM effect was observed in the lenalidomide-insensitive cell line JJN3 (Figure 6A). In addition, we tested this combination approach in primary MM cells from 18 patients and observed that the apoptosis rate increased by 20% (Figure 6C).

### 2.8. Carfilzomib-Induced Apoptosis Is Strongly Increased by Concomitant Treatment with Ancistrocladinium A

To determine the potential combinatory effects of ancistrocladinium A and the clinically most effective PI carfilzomib, we combined both compounds at their respective EC_25_-concentration levels for 3 d prior to assessing cellular viability. Whereas the combination efficiently worked in JJN3 and MM1.S cells, it did not lead to increased apoptosis levels in INA-6 cells (Figure 6B). However, viability analyses of 18 primary MM cell samples revealed that concomitant treatment of ancistrocladinium A significantly increased carfilzomib-induced apoptosis by 22% (Figure 6D).

## 3. Discussion

As most MM patients still succumb to their disease, the development of therapeutic strategies remains a scientific challenge. Our study provides the first experimental evidence that the natural alkaloid ancistrocladinium A exhibits anti-MM activity even in PI-resistant MM by targeting the RNA binding and splicing machinery of the MM cells, leading to DNA damage and apoptosis.

Ancistrocladinium A belongs to the class of *N,C*-coupled naphthylisoquinoline alkaloids and is equipped with an unprecedented chiral *N*-iminium aryl axis and a permanent positive charge. It has gained increasing attention because of its remarkable biological activities [23]. In eight MM cell lines (except for AMO1), two Ixa-resistant cell lines, and the lenalidomide-insensitive cell line JJN3, we observed apoptosis upon ancistrocladinium A treatment. CFZ-resistant cell lines, on the contrary, were almost insensitive toward ancistrocladinium A, suggesting that ABCB1 [9] and ABCB4 genes (MDR3) as underlying resistance mechanisms lead to flush-out of ancistrocladinium A [9]. However, resistance mechanisms to bortezomib and ixazomib, based on PSMB5 overexpression or mutation of PSMB5 [10,11], could be overcome by ancistrocladinium A, indicating a mechanism independent of the PI-binding site of PSMB5. In addition to cell line models, which might differ from the patient’s biology in vivo, we found strong induction of apoptosis by ancistrocladinium A in primary MM cells freshly isolated from the patient’s BM specimen in the presence of protective primary BMSCs. In contrast, primary non-malignant PBMCs were completely insensitive to ancistrocladinium A. Taken together, our data indicate an anti-tumor-specific apoptotic effect of ancistrocladinium A on MM cells.

The labeling of small bioactive organic molecules with biotin, followed by affinity chromatography, has become an attractive technique for the identification of their target proteins and, thus, for the elucidation of the respective mode of action and metabolic pathways [27,28]. Here we have synthesized, for the first time, biotinylated ancistrocladinium A (Figure 1D) to pull down the interacting proteins or protein complexes prior to detection using an MS. We identified 79 proteins that might interact with ancistrocladinium A directly or through a protein complex. The data suggest that ancistrocladinium A might bind to multiple proteins. Interestingly, most of these proteins belong to RNA binding and RNA splicing-associated proteins. These MS data gave a clear hint that ancistrocladinium A might regulate the gene expression and alternative splicing of RNA by direct binding or modulating the cascade of these processes. This is the first report that provides insight into the mode of action of *N,C*-coupled NIQ alkaloids.

To further confirm that ancistrocladinium A targets RNA processes like RNA binding, splicing, and transcription, we performed a gene expression and splicing profiling. Indeed, 1103 genes, including pseudogenes, were upregulated, and 184 genes and pseudogenes were downregulated after treatment with ancistrocladinium A. The enriched proteins include various splicing factors like SRSF1, which had previously been shown to be an essential driver for MM [17] or SF3B1, where it was proven in chronic lymphatic leukemia (CLL) that mutations in the protein directly affect the DNA damage response [29]. In addition, we found various genes and pseudogenes encoding for the spliceosomal complex, like U1, U2, U4, U5, and U6, to be differentially regulated. Furthermore, 57 small nucleolar RNAs (snoRNAs) were found to be differentially regulated, strengthening alternative pre-mRNA splicing as a potential functional target. SnoRNAs are non-protein coding RNAs that guide post-transcriptional modifications of ribosomal and small nuclear RNAs [30,31] or, like SNORD116, regulate alternative splicing [32]. Another set of genes with expression changes belongs to small Cajal body-specific RNAs (scaRNAs), which have a specific role in spliceosome formation and ribosome maturation and facilitate modifications of U1, U2, U4, and U5 snRNAs [33]. The expression changes in these genes and the top enriched pathways strengthen the finding that ancistrocladinium A targets pre-mRNA splicing as indicated by the enrichment of various splicing factors. RNA splicing and transcriptions are crucial processes for cell survival and growth. Tumor cells normally have a high turnover of RNA and proteins, thereby growing much faster than normal cells [34]. Therefore, the concept of using RNA splicing-inducing compounds as a therapy has been investigated for a long time, and different inhibitor classes have been characterized during the past years. For example, the CLK inhibitor TG003 inhibits CLK1, CLK2, and CLK4 by competitively binding to the ATP binding site, thereby causing the dissociation of nuclear speckles, splicing modulation, and expression of transcripts that encode for SRSF2 and CLK kinases [35]. Another class is herboxidienes, which decrease the number of nuclear speckles but increase their size [36,37]. The class of spliceostatins includes meayamycin B, spliceostatin A, and different variants of sudemycin causing cell cycle arrest or an alternative, functionally intact but degradation-deficient p27 isoform [15,38,39]. Our study adds ancistrocladinium A to the list of anti-cancer agents that targets the RNA transcription and splicing machinery in tumor cells.

Genes differentially regulated by ancistrocladinium A include pathways associated with the transcription initiation factors IIA Gamma Chain (GTF2A2) and the IIE subunit beta (GTF2E2). Both GTF2A2 and GTF2E2 are known to be involved in the initiation of RNA transcription and are frequently overexpressed in cancer [40,41]. The results support the data of MS analysis and confirm that RNA transcription-associated proteins were enriched due to these pathways. Further, many genes of the RNA processing pathway were differentially expressed, thus confirming previous MS data where we had found the RNA polymerase ε to be one potential target protein. In addition, we identified PSMB5 target gene regulation in response to ancistrocladinium A treatment. The proteasome subunit PSMB5 is a key protein for MM as it harbors the binding site for the proteasome inhibitors carfilzomib, ixazomib, and bortezomib [4]. The Gene Transcription Regulation Database (GTRD) determined the target genes of PSMB5 based on at least one transcription motif in the promotor region of the respective gene [42]. These genes include various histones, AFT4, and different heat shock proteins such as HSP90, HSP70, or HSP40 that are involved in different cellular stress response mechanisms. These data further strengthen our assumption that ancistrocladinium A-induced apoptosis is mediated by triggering cellular stress.

We have shown caspase-dependent PARP-1 cleavage in the ancistrocladinium A-sensitive MM cell lines but not in insensitive AMO-1 cells. The different sensitivity for ancistrocladinium A treatment might be explained, at least in part, by different stress response patterns. Whereas the ancistrocladinium A-sensitive INA-6 and MM.1S cells showed both induction of proteotoxic and oxidative stress response pathways (by ATF4 and ATM/H2A.X), AMO-1 cells almost lacked oxidative stress induction by the ATM/H2A.X pathway. ATF4 has a dual function as it can be protective under proteotoxic stress, or it can induce cell death if the stress exceeds the protective capacity [43]. Thus, we could show that ixazomib-treatment of PI-sensitive MM cell lines resulted in UPR-induced ATF4 expression and apoptosis [11]. Elevated ATF4 can also trigger oxidative stress and induce ferroptosis, a regulated cell death that is initiated by the generation of reactive oxygen species (ROS), deregulated iron metabolism, and accumulation of lipid peroxidation products [44]. In addition, ATF4 has also been described as a key regulator of the mitochondrial stress response, which is linked to oxidative stress and to apoptosis regulation [45]. Histones like H2A.X have different roles in the cells depending on the cellular context, which is regulated via the induction of phosphorylation. Thus, it contributes to the remodeling of the DNA into chromatin [46] and induction of DNA damage response [47], but also mediates oxidative stress response independent of DNA damage. H2A.X activity is regulated either by the serine/threonine kinase ataxia-telangiectasia mutated (ATM) or the Rad3-related protein (ATR) [48]. We observed that H2A.X phosphorylation mainly occurred in ancistrocladinium A-sensitive cells, suggesting that in addition to proteotoxic stress, induction of oxidative stress might be critical for the ancistrocladinium A-induced apoptotic effect. In contrast to ATM, the ATR-p53 axis was not induced as there were no expression changes on whole ATR or phosphorylation levels, and downstream of ATR, p53 expression was rather decreased. This indicates that potential apoptotic mechanisms are regulated independently of an ATR-p53-driven DNA damage response [49].

Proteasome inhibitors like bortezomib, ixazomib, and carfilzomib have been successfully established as powerful therapeutic agents for the treatment of MM patients [50]. However, during the course of the disease, MM patients frequently develop resistance to PI therapy. Therefore, combination approaches are needed to improve the therapeutic effect of PI leading to prolonged survival of patients. Thus, treatment with the HDAC inhibitor panobinostat, which also induces proteotoxic stress, enhances the apoptotic effects of bortezomib and is clinically approved as a PI combination partner [11]. Here we demonstrate that concomitant ancistrocladinium A treatment strongly enhanced the apoptotic effect of either carfilzomib or panobinostat in MM cell lines and in primary MM cells. As discussed above, we found that ancistrocladinium A alone induces proteotoxic and oxidative stress, which might synergize with PI-/HDAC-induced proteotoxicity resulting in a more pronounced apoptotic effect. Thus, it has been described that proteotoxic stress can trigger oxidative stress and, vice versa, exceeding the capacity of protective stress response mechanisms to keep homeostasis [3]. Many adaption mechanisms that might contribute to the development of PI resistance have been described, including overexpression or point mutations within the PI-binding site of the proteasome subunit PSMB5 [11,51]. In addition, overexpression of the ABC transporter ABCB1 (alternative name: multi-drug resistance protein 1, MDR1) [9] was observed as a resistance mechanism. To successfully treat relapses in such a PI-resistant state, the development of alternative strategies to overcome or circumvent PI resistance might represent a suitable strategy.

In summary, the natural product ancistrocladinium A might act as a potential anti-myeloma drug alone or in combination with proteasome or HDAC inhibitors. Our preliminary data show that it targets the spliceosomal complex and induces alternative splicing, but further investigations are required to understand its detailed mode of action.

## 4. Materials and Methods

### 4.1. Pharmacological Inhibitors

All inhibitors were dissolved in H_2_O-free DMSO and stored at the indicated stock concentrations. Carfilzomib (20 mM) and panobinostat (10 mM) were from Selleck Chemicals (Houston, TX, USA), and ancistrocladinium A (10 mM) was prepared as described before [52]. Working solutions were prepared freshly from the stock solutions in the cell culture medium.

### 4.2. Synthesis of Biotinylated Ancistrocladinium A

A de novo synthesis of biotinylated ancistrocladinium A was performed based on a strategy similar to the one following the given previously published synthetic pathways to the natural alkaloid ancistrocladinium A [53]. Details are given in the Section 2.1 and Section 2.2 and in Supplementary Figure S1. Biotinylated ancistrocladinium A (Figure 1D) was used to pull down the interacting proteins or protein complexes using streptavidin beads (Figure 2B) and identified them using an MS-based approach.

### 4.3. MS-Based Spectroscopy and Data Analysis

The MS-based drug target identification and data analysis are described in the Section 2.1 and Section 2.2, and a schematic overview of the experiment is shown in Supplementary Figure S2.

### 4.4. Cell Culture

The cell lines RPMI-8226, KMS12 BM, JJN3, KMS11, NCI-H929, OPM2, MM1.S, INA-6, AMO-1, L363, the ixazomib-resistant sub-cell lines AMO1R180Ixa, MM1.SR180Ixa, and L363R350Ixa [8], as well as the carfilzomib-resistant sub-cell lines AMO1R65Car and INA6R17Car [54] were used in this study. Cell culture was performed as described before [54,55]. Peripheral blood mononuclear cells (PBMCs) were obtained from buffy coats of healthy donors after Ficoll density centrifugation and cultured in RPMI-1640 medium (Sigma-Aldrich, St Louis, MO, USA) supplemented with 10% FBS (Sigma-Aldrich), 100 U/mL penicillin/100 µg/mL streptomycin (Sigma-Aldrich), 2 mM L-glutamine (Sigma-Aldrich), 1 mM Na-pyruvate (Sigma-Aldrich), and 10 mM HEPES (Sigma-Aldrich).

### 4.5. Isolation of CD138+ Primary MM Cells

Primary MM cells and BMSC (bone marrow stroma cells) were obtained from diagnostic bone marrow (BM) aspirates of MM patients after obtaining informed consent with permission of the Ethics Committee of the University of Würzburg (76/13). Isolation and cultivation of CD138+ MM cells were performed as described before [56]. Relevant patients’ information regarding age, sex, therapies, known PI resistances, and known mutations is summarized in Supplementary Table S4.

### 4.6. Viability Analysis

For viability analysis, 7500–10,000 cells of the respective MM cell lines or 50,000 PBMCs were seeded per well of a 96-well plate, and different concentrations of ancistrocladinium A or combination approaches using EC_25_ concentrations of ancistrocladinium A and either carfilzomib or panobinostat followed by a flow cytometry-based annexin V/propidium iodide staining after 3 d incubation time. For primary MM cells, 10,000 were seeded on bone marrow stroma cells (BMSCs) and treated with Ancistro or the combination approaches of ancistrocladinium A and carfilzomib or panobinostat accordingly. After 3 d, only annexin V negative, propidium-iodide negative cells were considered for viability analysis. Data were normalized against DMSO. A minimum of three independent experiments were performed, and calculation of mean ± standard deviation and curves plotting was conducted in Prism V. 7.04 (GraphPad, GraphPad Software, La Jolla, CA, USA).

### 4.7. RNA Isolation

INA-6 cells (1 × 10^6^ in 3 mL RPMI-1640 complete medium) were treated with 3 µM ancistrocladinium A for 4 h following RNA isolation was performed using a PEQgold RNA extraction kit (Peqlab, Erlangen, Germany) according to the manufacturers’ guidelines.

### 4.8. GeneChip Microarray Assay

Sample preparation for microarray hybridization was carried out as described in the Affymetrix GeneChip WT PLUS Reagent Kit User Manual (Affymetrix, Inc., Santa Clara, CA, USA).

In brief, 200 ng of total RNA was used to generate double-stranded cDNA. Subsequently, synthesized cRNA (15 µg) was purified and reverse transcribed into sense-strand (ss) cDNA, into which unnatural dUTP residues were incorporated. Purified ss cDNA was fragmented using a combination of uracil DNA glycosylase (UDG) and apurinic/apyrimidinic endonuclease 1 (APE 1), followed by terminal labeling with biotin. Then, 5.5 µg fragmented and labeled ss cDNA were hybridized to Affymetrix Clariom D human arrays for 16 h at 45 °C in a GeneChip hybridization oven 640. Hybridized arrays were washed and stained in an Affymetrix Fluidics Station FS450, and the fluorescent signals were measured with an Affymetrix GeneChip Scanner 3000 7G. Fluidics and scan functions were controlled by Affymetrix GeneChip Command Console v4.1.3 software.

Sample processing was performed at an Affymetrix Service Provider and Core Facility, “KFB—Center of Excellence for Fluorescent Bioanalytics” (Regensburg, Germany; www.kfb-regensburg.de, accessed on 20 November 2017).

### 4.9. Microarray Data Analysis

Summarized probe set signals in the log2 scale were calculated using the GCCN-SST-RMA algorithm with the Affymetrix GeneChip Expression Console v1.4 software. After exporting into Microsoft Excel, average signal values, comparison fold changes, and significance *p*-values were calculated. Probe sets with a fold change above 2.0-fold and a Student’s *t*-test *p*-value lower than 0.05 were considered significantly regulated. Data are available online with Geo-accession number: GSE236602.

Splicing expression changes were calculated using TAC4.0 software (Affymetrix; Thermo Fisher Scientific, Inc., Santa Clara, CA, USA). Filter criteria for splicing expression changes were that genes expressed in both conditions were considered as true, the exon splicing index had to be ≥2 or ≤2, the exon *p*-value < 5%, and the exon had to be expressed under at least one condition.

### 4.10. Gene Set Enrichment Analysis

A further gene set enrichment analysis (GSEA) was performed for RNA expression and RNA splicing data using the GSEA software V4.1.0 [57,58]. Briefly, the tables of RNA expression and RNA splicing data were provided to the software, and analysis was run to identify pathways where the genes are involved.

### 4.11. Western Blot Analysis

INA-6, MM1.S, and AMO1 cells were treated with ancistrocladinium A for 4 h, followed by protein lysis as described before [54]. Western blotting was performed as reported earlier [11] using the antibodies listed in Supplementary Table S5.

## 5. Conclusions

In summary, the natural product ancistrocladinium A is a promising novel anti-myeloma drug candidate for further translational development. Although its precise mechanism of action has to be elucidated in further studies, our preliminary data show that it targets the spliceosomal complex and induces alternative splicing leading to induction of cellular stress and apoptosis also in PI-resistant MM, in particular in combination with proteasome or HDAC inhibition.

## Figures and Tables

**Table 1:** Top 10 pathways with gene expression changes. Shown are the pathways of the strongest differentially expressed genes after a gene set enrichment analysis (GSEA). The number of upregulated and downregulated genes per pathway is written and visualized (green: upregulated, red: downregulated) with bars.

Gene Set Name [# Genes (K)]	# Genes in Overlap (k)	k/K	*p*-Value	fdr q-Value	Gene Regulation
GOBP_RNA_PROCESSING [1424]	102	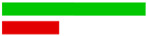	3.13 × 10^−29^	1.01 × 10^−24^	73 upregulated, 29 downregulated
GTF2A2_TARGET_GENES [522]	50		6.23 × 10^−20^	1.01 × 10^−15^	46 upregulated, 4 downregulated
SNAPC4_TARGET_GENES [138]	27	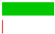	3.3 × 10^−19^	3.55 × 10^−15^	26 upregulated, 1 downregulated
GOBP_FORMATION_OF_QUADRUPLE_SL_U4_U5_U5_U6_SNRNP [10]	10	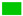	9.08 × 10^−18^	7.33 × 10^−14^	10 upregulated, 0 downregulated
GOCC_NUCLEOLUS [1386]	76		2.17 × 10^−15^	1.4 × 10^−11^	54 upregulated, 22 downregulated
ZHENG_CORD_BLOOD_C5_SIMILAR_TO_HSC_C6_C6_PUTATIVE_ALTERED_METABOLIC_STATE [97]	20	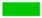	4.73 × 10^−15^	2.54 × 10^−11^	20 upregulated, 0 downregulated
GTF2E2_TARGET_GENES [411]	38	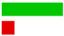	5.87 × 10^−15^	2.71 × 10^−11^	32 upregulated, 6 downregulated
GOBP_SPLICEOSOMAL_TRI_SNRNP_COMPLEX_AS_ASSEMBLY [25]	12	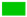	1.43 × 10^−14^	5.78 × 10^−11^	12 upregulated, 0 downregulated
GOCC_SMALL_NUCLEAR_RIBONUCLEOPROTEIN_CN_COMPLEX [99]	19	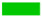	9.09 × 10^−14^	3.26 × 10^−10^	19 upregulated, 0 downregulated
GOBP_RIBONUCLEOPROTEIN_COMPLEX_BIOGENEENESIS [471]	38	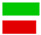	4.27 × 10^−13^	1.38	20 upregulated, 18 downregulated

**Table 2:** Top 10 pathways of genes with splicing alterations. Shown are the top 10 pathways of the genes with splicing alterations after a gene set enrichment analysis (GSEA).

Gene Set Name [# Genes (K)]	# Genes in Overlap (k)	k/K	*p*-Value	FDR q-Value
PSMB5_TARGET_GENES [307]	68	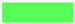	3.31 × 10^−63^	1.07 × 10^−58^
GTF2A2_TARGET_GENES [522]	69	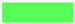	3.43 × 10^−48^	5.54 × 10^−44^
SETD7_TARGET_GENES [991]	83	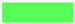	2.09 × 10^−42^	2.25 × 10^−38^
FISCHER_DREAM_TARGETS [968]	81	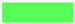	2.43 × 10^−41^	1.96 × 10^−37^
REACTOME_CELLULAR_RESPONSES_TO_EXTERNARNAL_STIMULI [706]	70	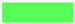	1.81 × 10^−40^	1.17 × 10^−36^
BARX2_TARGET_GENES [1723]	101	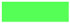	4.94 × 10^−38^	2.66 × 10^−34^
REACTOME_CELL_CYCLE [693]	66	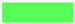	4.41 × 10^−37^	2.03 × 10^−33^
ZNF84_TARGET_GENES [2000]	107	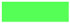	7.93 × 10^−37^	3.2 × 10^−33^
REACTOME_CELL_CYCLE_MITOTIC [561]	60	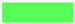	1.39 × 10^−36^	4.98 × 10^−33^
MARTENS_TRETINOIN_RESPONSE_DN [839]	70	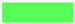	1.28 × 10^−35^	4.14 × 10^−32^

## Data Availability

RNA array data can be found at https://www.ncbi.nlm.nih.gov/geo (accession no: GSE236602) and MS data on the PRIDE database https://www.ebi.ac.uk/pride/ (PXD043261).

## Supplementary Materials

The following supporting information can be downloaded at: https://www.mdpi.com/article/10.3390/ph16081181/s1, Supplementary Figure S1: Overview of the synthesis of the biotin-labeled ancistrocladinium A derivative 2b. Supplementary Figure S2: Affinity capture experiment for identification of potential target proteins and their interaction partners of ancistrocladinium A. Supplementary Figure S3: Ancistrocladinium A induces gene expression alterations and alternative splicing. Supplementary Figure S4. Ancistrocladinium A decreases the cells in G0/G1 phase. Supplementary Figure S5: Ancistrocladinium A induces ATF4 protein expression in INA-6 cells. Supplementary Figure S6: Ancistrocladinium A induces PARP-1 cleavage. Supplementary Table S1: List of enriched proteins of 4’-biotinylated Ancistrocladinium A using MS approach. Supplementary Table S2: Differentially expressed genes. The table shows the differentially expressed genes of INA-6 cells after 4 h of treatment with 3 µM Ancistrocladinium A. Supplementary Table S3: Genes with transcriptional changes. This table shows the genes of INA-6 cells after 4 h treatment with 3 µM Ancistrocladinium A. Supplementary Table S4: Summary of patient characteristics and treatment schedules. Supplementary Table S5: Antibodies used for Western blots. Details of the antibodies that were used in the study.
