# Supplementary material for: Ancistrocladinium A Induces Apoptosis in Proteasome Inhibitor-Resistant Multiple Myeloma Cells: A Promising Therapeutic Agent Candidate
Source: Pharmaceuticals (Basel). 2023 Aug 18;16(8):1181. doi: 10.3390/ph16081181 (PMC10459547; doi:10.3390/ph16081181)
Supplement: Supplementary file 1 [file pharmaceuticals-16-01181-s001.zip › pharmaceuticals-2534250-supplementary/supplementary_files/Supplementary_Figures_legends.pdf]

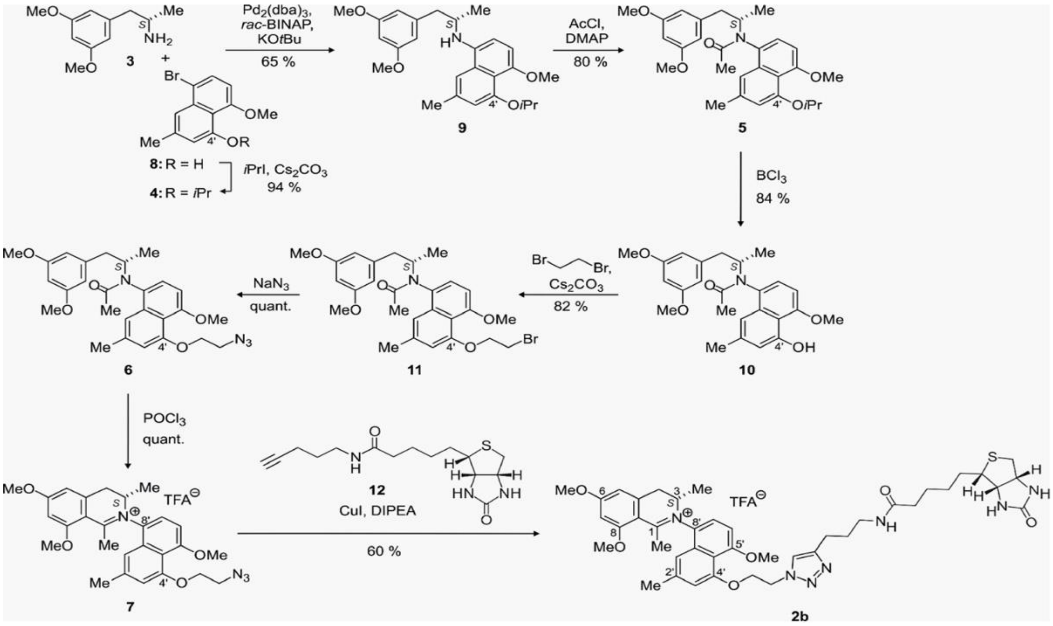

Figure S1. Overview of the synthesis of the biotin-labeled ancistrocladinium A derivative 2b.

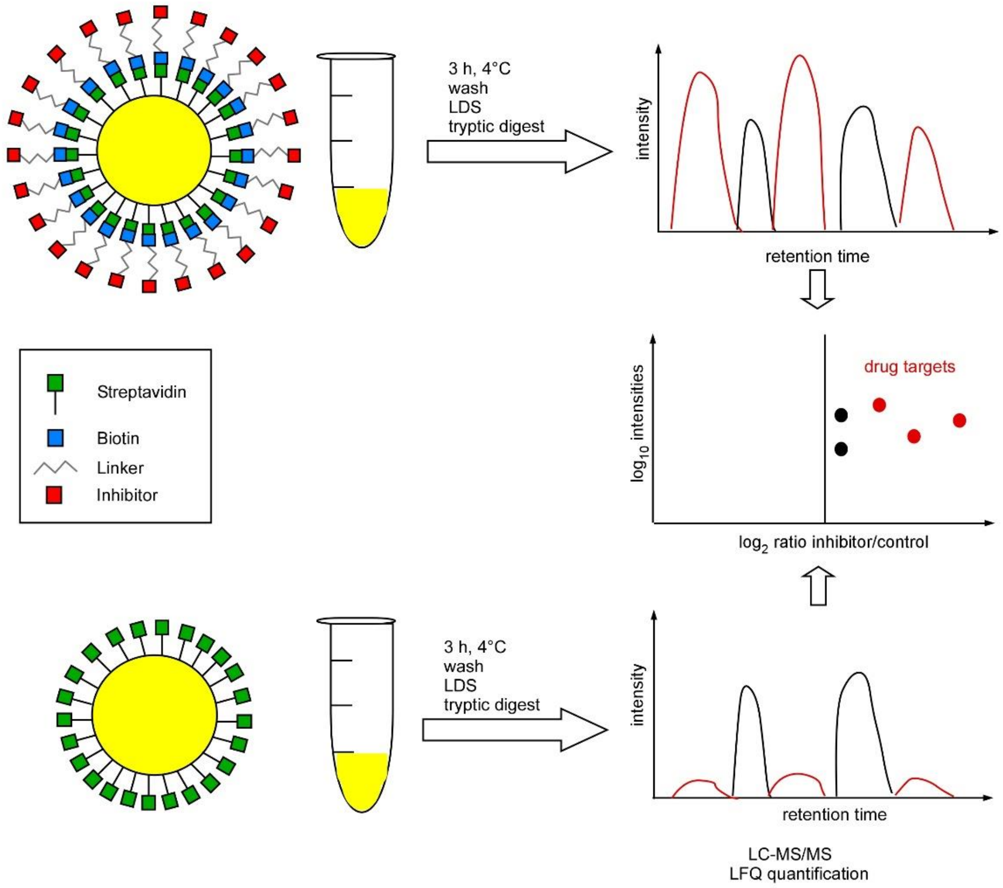

Figure S2. Affinity capture experiment for identification of potential target proteins and their interaction partners of ancistrocladinium A. A biotinylated inhibitor was loaded on streptavidin beads; empty beads were used as control. Both beads were incubated with INA-6 cell lysate for 3 h at 4 °C and washed, and captured proteins were eluted with LDS sample buffer. Proteins were digested with trypsin and analyzed by nanoLC-MS/MS. Label-free quantification (LFQ) of both samples was performed for all identified proteins. In the intensity vs. LC-MS/MS LFQ quantification, the different protein sequences with different abundancies are schematically drawn in 2

different colors (black = not specifically enriched proteins, red = specifically enriched proteins that bind to ancistrocladinium A). Specifically enriched drug target proteins are in drawn in red in the $\log_{10}$ intensities vs. $\log_2$ ratio inhibitor/control blot. Not significantly enriched proteins are drawn in black.

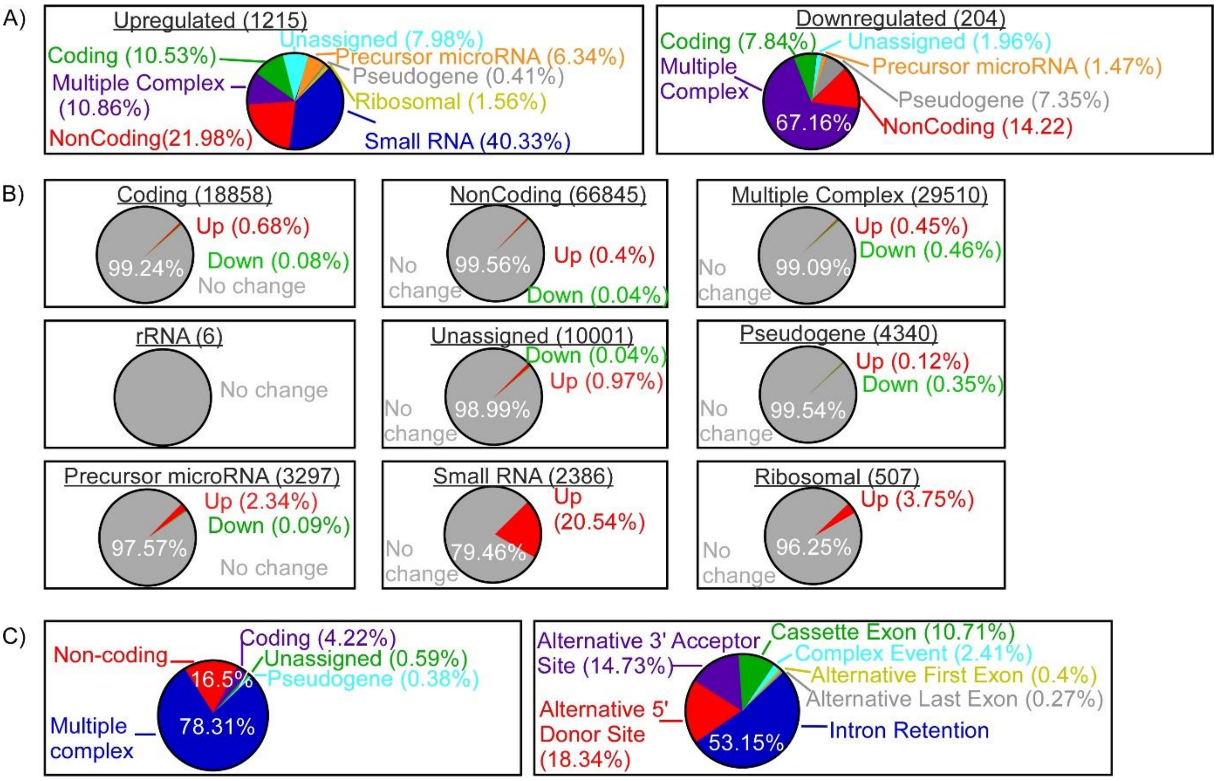

Figure S3. Ancistrocladinium A induces gene expression alterations and alternative splicing. INA-6 cells were treated for 4 h with 3 μM Ancistrocladinium A or DMSO as control followed by RNA extraction, cRNA synthesis, ss cDNA transcription and hybridization to Affymetrix Clarion D human array. Afterwards, (A & B) gene expression changes and (C) splicing expression alterations were determined.

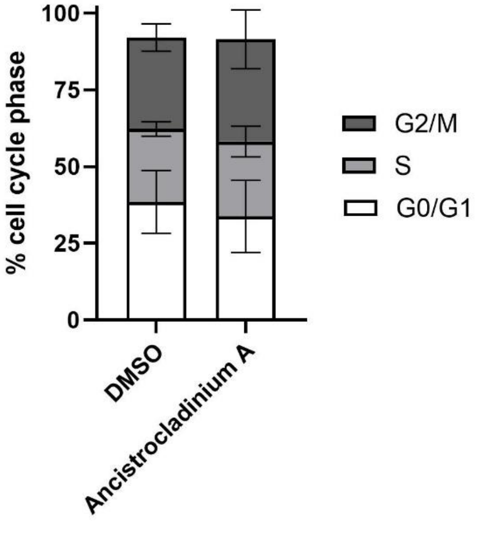

	DMSO	Ancistrocladinium A	
	mean ± SD	mean ± SD	paired t-test
G2/M	29.4 ± 4.5	33.3 ± 9.6	p=0.273049
S	23.8 ± 2.4	24.4 ± 5.0	p=0.745026
G0/G1	38.4 ± 10.2	33.8 ± 11.4	p=0.108137

Figure S4. Ancistrocladinium A decreases the cells in G0/G1 phase. INA-6 cells were treated for 24 h with 3 µM Ancistrocladinium A and followed by propidium iodide staining and flow cytometry-based cell cycle analysis. Four independent experiments were performed and a paired t-test with multiple comparisons was performed.

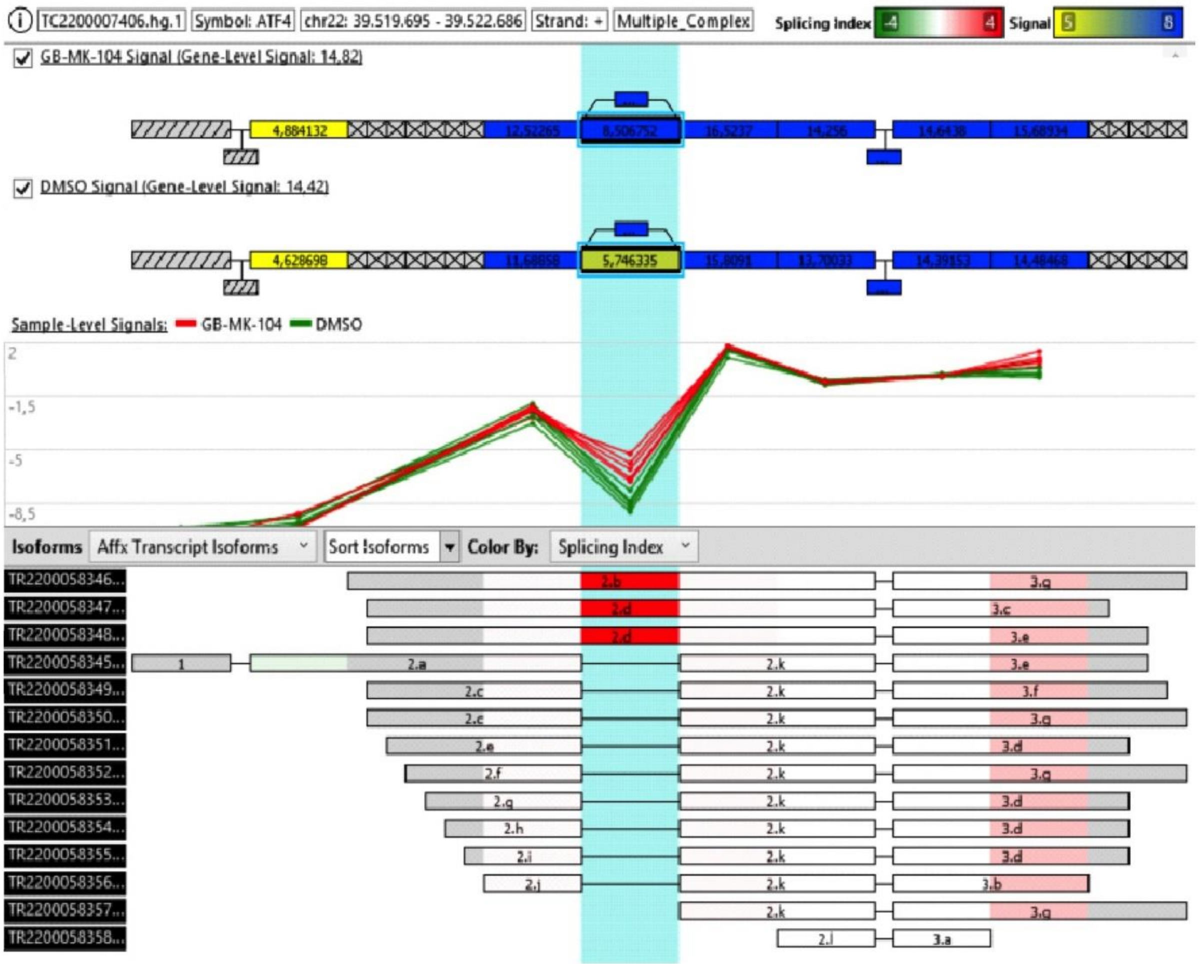

Figure S5. Ancistrocladinium A induces ATF4 protein expression in INA-6 cells. Splicing effect on ATF4-RNA 4 h after treatment of INA-6 cells with ancistrocladinium A (here labelled as GB-MK-104). Shown is the original low-resolution graph that was generated by the TAC4.0 software. Of note, in Figure 4, this graph was redrawn for better quality.

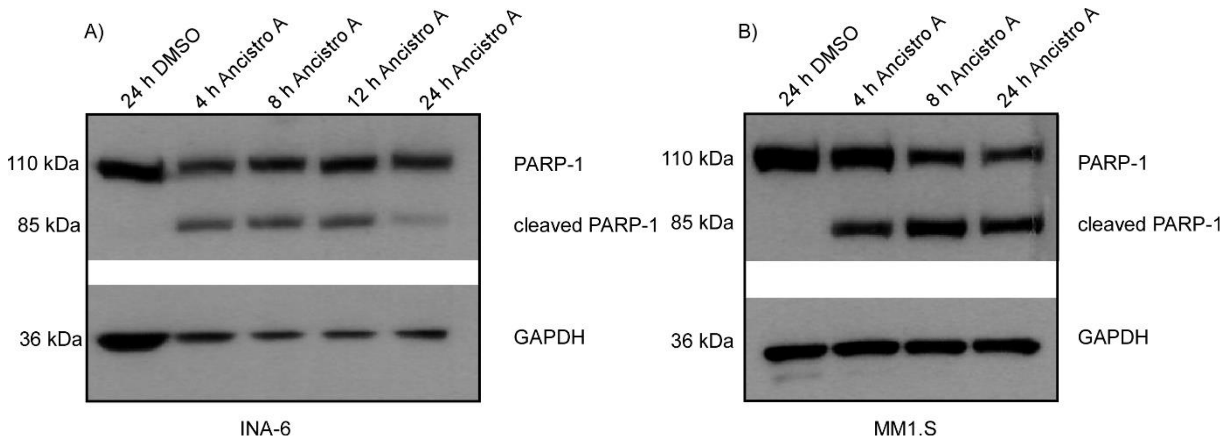

Figure S6. Ancistrocladinium A induces PARP-1 cleavage. **(A)** INA-6 and (B) MM1.S cells were treated for 4 h, 8 h, 24 h and INA-6 cells also for 12 h with 3 μM Ancistrocladinium A followed by cell lysis and Western blot analysis of PARP-1 cleavage. GAPDH served as loading control.
